# A 3D-Printed Standardized Modular Microfluidic System for Droplet Generation

**DOI:** 10.3390/bios12121085

**Published:** 2022-11-28

**Authors:** Junyi Chen, Shaoqi Huang, Yan Long, Kan Wang, Yangtai Guan, Lianping Hou, Bo Dai, Songlin Zhuang, Dawei Zhang

**Affiliations:** 1 Engineering Research Center of Optical Instrument and System, the Ministry of Education, Shanghai Key Laboratory of Modern Optical System, University of Shanghai for Science and Technology, Shanghai 200093, China; 2 Department of Neurology, Renji Hospital, School of Medicine Shanghai Jiaotong University, No. 160 Pujian Rd., Shanghai 200127, China; 3 James Watt School of Engineering, University of Glasgow, Glasgow G12 8QQ, UK

**Keywords:** modular microfluidics, droplet generation, standardized microfluidics

## Abstract

Droplet-based microfluidics has a variety of applications, such as material synthesis and single-cell analysis. In this paper, we propose a modular microfluidic system using projection micro-stereolithography three-dimensional (3D) printing technology for droplet generation. All modules are designed using a standard cubic structure with a specific leakage-free connection interface. Versatile droplets, including single droplets, alternating droplets, merged droplets, and Janus particles, have been successfully produced. The droplet size and the generation rate can be flexibly controlled by adjusting the flow rates. The influence of the flow rate fraction between the discrete phase and the continuous phase over the generation of the alternating and merged droplets is discussed. Furthermore, the ‘UV curing’ module can be employed to solidify the generated droplets to avoid coalescence and fix the status of the Janus particles. The proposed modular droplet generators are promising candidates for various chemical and biological applications, such as single-cell incubation, screening of protein crystallization conditions, synthesis of nanoparticles, and gene delivery. In addition, we envision that more functional modules, e.g., valve, microreactor, and detection modules, could be developed, and the 3D standardized modular microfluidics could be further applied to other complex systems, i.e., concentration gradient generators and clinical diagnostic systems.

## 1. Introduction

Microfluidic technique plays an important role in various chemical analyses and biomedical diagnoses, including nucleic acid analysis, clinical testing of viruses, single-cell proteomics, diagnosis of cancers, organ-on-a-chip, drug screening, and chemical compositional determination [1,2,3,4,5,6,7]. Recently, droplet microfluidics has been widely used in various applications due to its intrinsic advantages, e.g., high surface-area-to-volume ratio for fast reaction [8], high throughput [9], and low reagent usage [10]. In general, droplets can be generated in different forms. Single droplets have been widely used to encapsulate cells in picolitres for single-cell analysis [11]. Alternating droplets are formed by two or more kinds of single droplets that are arranged alternately in series. The alternating droplets can be used to screen conditions of protein crystallization, where the reaction is conducted in the foregoing droplet, and the second droplet is used to index the composition of the first droplet [12]. Droplets merged with two discrete phases can be used as a microreactor to mix reagents quickly and efficiently to synthesize nanoparticles with controllable fusion [13]. Double emulsions with three separate phases can be used for versatile targeted therapeutics and antibody combination therapy [14]. Janus particles are formed with two constituents and an interface exists to separate the constituents [15]. Janus particles can be used to deliver exogenous genes, where one half of the Janus particle immobilizes a targeting ligand while the other half immobilizes the nucleic acid [16].

Current techniques of droplet generation can be divided into two categories, i.e., active techniques that utilize external mechanisms to generate droplets and passive techniques that do not use any external actuation. Active droplet generation techniques employ various external mechanisms like electrical, magnetic, centrifugal, optical, thermal, and mechanical controls [17,18,19,20,21,22]. Although active droplet generation techniques have the advantage of flexibility in controlling droplet formation, harnessing external mechanisms makes the system complex and the operation complicated. Alternatively, passive techniques employing the geometry of microfluidics exhibit a convenient and low-cost method for droplet generation. Cross-flow, co-flow, and flow-focusing structures are three major geometries for passive droplet generation. All kinds of droplets can be generated in these geometries. For example, a double T-junction, as a cross-flow geometry, can be used to generate alternating droplets and merged droplets.

Modular microfluidic technology paves a new way in the microfluidic fields [23,24,25]. An entire microfluidic system is divided into multiple sub-systems, called modules. Each module has a specific function. In comparison to a monolithic microfluidic system, a modular system has high flexibility in function because the key parts can be easily replaced, and the system can be expanded. Recently, modular microfluidics has been applied to droplet generation [26,27,28,29]. Thanks to the advancement of 3D-printing techniques, various modular microfluidic droplet generation systems have been developed based on 3D-printed modules. One strategy is to design modules to equip auxiliary components, including needles and glass capillaries, for droplet generation [26,27]. Since the inner diameters of the glass capillaries and the needles are very small, it is feasible to generate small, stable droplets. The modules are designed to match the structures of the auxiliary components. The modules are used to install the auxiliary components and are built into a whole droplet generation system. However, employing non-standard customized auxiliary components might cause problems of instability and inconsistency. Alternatively, the modules containing the structures of mixers and nozzles can be directly produced by 3D printing [28,29]. The modules with different functions can be connected to form an entire microfluidic droplet generation system. Nevertheless, there is no standard to regulate the design and the use of modules. The non-uniform design restricts the assembly of the modules, limiting the extensibility of the system. Thus, it is preferred to use standard modules so that the modules can be freely modified, and the system can be easily updated.

In this paper, we demonstrate droplet generation by using a 3D-printed modular microfluidic system. A standard for designing and utilizing modules and building microfluidic systems is established. The size of the module, the structure of microchannels, and the alignment and connection methods are stipulated in the standard. Thanks to the 3D-printing technique, fast design and cost-effective fabrication of the standard microfluidic modules are allowed. In addition, the 3D feature could also enrich the functions of the modules. A group of modules with specific functions is developed. The modules can be flexibly assembled to form droplet generators to produce different kinds of droplets. The droplet generators are compact, and the operation is simple. A variety of droplets, including single droplets, alternating droplets, merged droplets, and Janus particles, can be generated. The proposed modular droplet generator has the potential to be integrated into versatile droplet-based microfluidic systems.

## 2. Materials and Methods

### 2.1. Design and Fabrication of Droplet Generation Modules

**Figure 1:**
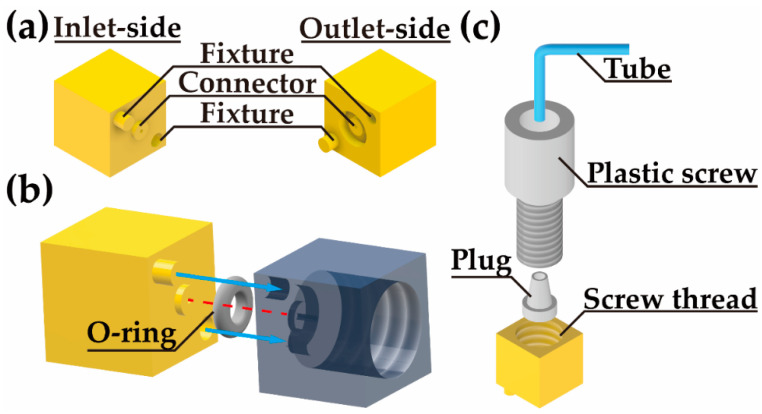
Schematics of the standard droplet generation modules. (**a**) Inlet and outlet sides of the standard modules. (**b**) The connection of the two modules. (**c**) Schematic diagram of the installation of the tube.

Droplet generation modules are designed with a standard cubic geometric structure, as shown in Figure 1. All the modules are in the uniform size of 8 × 8 × 8 mm^3^. On the inlet side and outlet side, there is an alignment and fixture structure. A short pillar and a shallow pit are on the two diagonal corners on the inlet side (Figure 1a), which are used to correspondingly match the pit and the pillar on the outlet side for the module-to-module assembly. At the center of the inlet side, a fluidic connector bulges out, while the connector is sunken on the outlet side. There is a groove around the fluidic connector on the outlet side.

As shown in Figure 1b, the inlet side can be inserted into the outlet side. The red dashed line shows the main channel of the interconnection between the modules. The size of the channel is 400 μm in diameter. A silicone O-ring with an inner diameter of 4 mm and a thickness of 1 mm is embedded in the groove on the outlet side. When the modules are assembled, the O-ring is compressed tightly to seal the gap to avoid the leakage of the fluids. Based on the standard structural design and connection method, any two modules can be flexibly and easily assembled.

Figure 1c shows the connection of the tube to the inlet module. The tube is threaded through a plastic screw and aligned with the channel of a plug. The plug is placed in the hole of the inlet module. The hole has a screw thread, matching that of the plastic screw. When the plastic screw is slowly screwed into the hole, the plug is squeezed by the screw. The stress applied to the plug shrinks the channel of the plug and tightens the tube. The fluid can then fluently enter the module and no leakage at the connector occurs.

**Figure 2:**
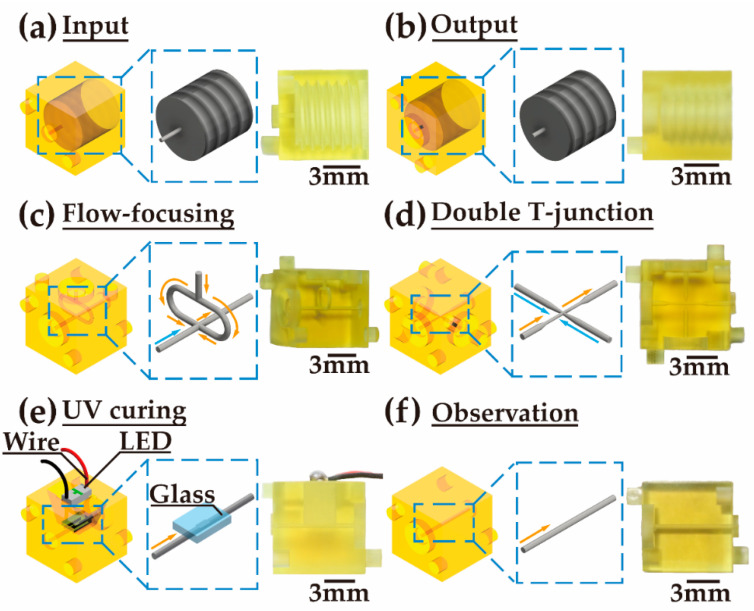
Schematic illustration of droplet generation modular components. (**a**) Input module. (**b**) Output module. (**c**) Flow-focusing module. (**d**) Double T-junction module. (**e**) UV curing module. (**f**) Observation module.

Various modules with specific functions are designed based on the standard mentioned above. The ‘input’ module is designed to feed the fluid into the module system from an external system via a tube (Figure 2a). The ‘output’ module, used to expel the fluid from the module system, has an opposite structure to that of the ‘input’ module (Figure 2b). The channel for the inlet and outlet is 400 μm in diameter.

A ‘flow-focusing’ module has two inlet sides and one outlet side (Figure 2c). The main channel connects the inlet side and the outlet side, which are opposite to each other. Another channel connecting to the other inlet side is split into two, and then the two branches join the main channel from the two sides. The cross-section of both channels is 400 μm in diameter. A ‘double T-junction’ module has three inlet sides and one outlet side (Figure 2d). One of the inlet sides is opposite to the outlet side. The other two inlets are on opposite sides. The main channel connecting the inlet and the outlet gradually shrinks from 400 μm to 200 μm in diameter in the middle of the channel. Two channels from the other two inlets connect to the center of the main channel from the two sides. The diameter of the two channels shrinks from 400 μm to 80 μm.

A ‘UV curing’ module is designed to solidify UV-curable material in the channel (Figure 2e). A straight channel connects the inlet side and the outlet side. The channel is open on one side in the middle. Fused silica glass is placed on the channel to seal the open side. An LED with a peak wavelength of 365 nm and a power of 60 mJ/s is installed above the glass.

In an ‘observation’ module, a straight channel connects the inlet and outlet sides (Figure 2f). The side of the module is transparent. The inside of the channel can be observed clearly. This module can be used to exhibit the droplets generated in the module system.

The 3D droplet generation modules are first designed by using computer-aided design software (Autodesk Inventor 2017). Then, the modules are fabricated by using a projection micro-stereolithography 3D printer (nanoArch^®^ P140, BMF Precision Technology Co., Chongqing, China). The feature size of the printing is 10 μm. UV-curable diacrylate polymer (HTL, BMF Precision Technology Co., Chongqing, China) is utilized as the 3D-printing material. The elastic modulus is about 3 GPa. The high flexural strength (112.9 MPa) and flexural modulus (1.8 GPa) allow the modules to be flexibly assembled. The 3D-printed microchannels have hydrophobic surfaces. To modify the wettability of the microchannels, hydrolyzed ethyl silicate solution (N-103X, Colcoat Co., Tokyo, Japan) [30] is injected into the channel, and the modules are heated at 120 °C for 30 min to vaporize the solvent.

### 2.2. Materials for Droplet Generation

The oil phase is prepared by dissolving 4.5% Span 80 (no. S6760, Sigma-Aldrich, Darmstadt, Germany), 0.40% Tween 80 (no. S-8074, Sigma-Aldrich, Darmstadt, Germany), and 0.05% Triton X-100 (no. T9284, Sigma-Aldrich, Darmstadt, Germany) in mineral oil (no. M-3516, Sigma-Aldrich, Darmstadt, Germany) [31] for the generation of single droplets, alternating droplets, and merged droplets. The deionized (DI) water mixed with green solvent dye (Yu Qian Building Materials Co., Shanghai, China) is used as the aqueous phase for the generation of single droplets. The DI water with an 80% wt/wt concentration of glycerol (no. G9012, Sigma-Aldrich, Darmstadt, Germany) is used as an aqueous phase for the generation of alternating droplets and merged droplets. Red dye (Yu Qian Building Materials Co., Shanghai, China), acting as an indicator, is dissolved in the DI water.

For the UV curing of single droplets, a mixture of pentaerythritol triacrylate (Aladdin Holdings Group Co., Beijing, China) (45.0 wt%), poly(ethylene glycol) diacrylate (Aladdin Holdings Group Co., Beijing, China) (45.0 wt%), acrylic acid (Aladdin Holdings Group Co., Beijing, China) (5.0 wt%), and 5.0 wt% of 1-hydroxycyclohexyl phenyl ketone (no. 405612, Sigma-Aldrich, Darmstadt, Germany) colored in red is used as the discrete phase. The required photocuring dose is about 2 J/cm^2^. In addition, a 2.0 wt% solution of sodium dodecylsulfate (SDS) (Aladdin Holdings Group Co., Beijing, China) is used as the continuous phase.

In the generation of Janus droplets, methacryloxypropyl dimethylsiloxane (Gelest, lnc., Morrisville, US) is used as one of the discrete phases in red, and a mixture of pentaerythritol triacrylate (Aladdin Holdings Group Co., Beijing, China) (45.0 wt%), poly(ethylene glycol) diacrylate (Aladdin Holdings Group Co., Beijing, China) (45.0 wt%), and acrylic acid (Aladdin Holdings Group Co., Beijing, China) (5.0 wt%) in green is used as another discrete phase. Both reagents contain 5.0 wt% of 1-hydroxycyclohexyl phenyl ketone (no. 405612, Sigma-Aldrich, Darmstadt, Germany) [32]. As well, 2.0 wt% SDS is used as the continuous phase. All reagents are freshly prepared for each experiment.

## 3. Results and Discussion

### 3.1. Generation of Single Droplets

**Figure 3:**
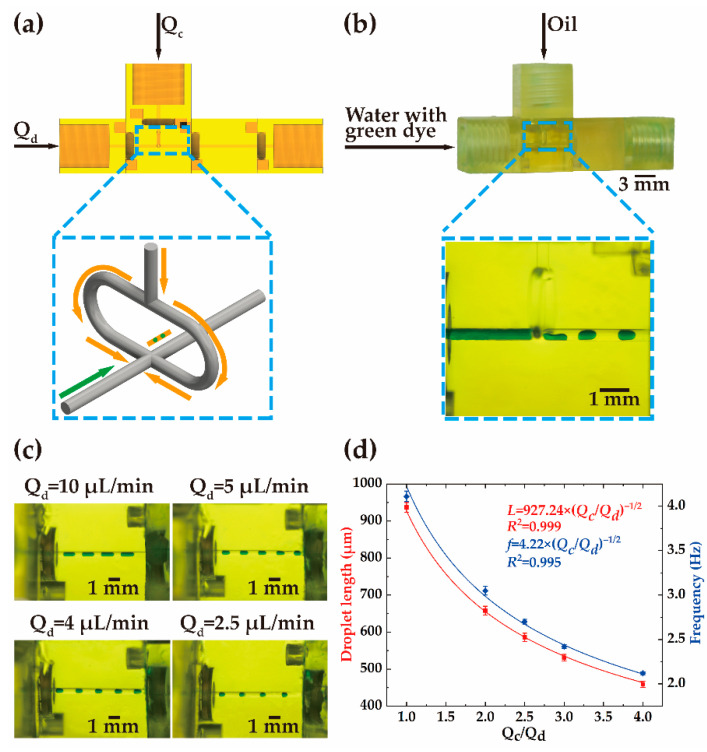
Schematic illustration of single-droplet generation. (**a**) Schematic and (**b**) the printed sample of assembled passive single-droplet generation device, respectively. (**c**) Optical micrographs of the produced single droplet at four flow rates of the discrete phase with the same flow rate (10 μL/min) of the continuous phase. (**d**) The plot of the flow rate ratio (*Q_c_*/*Q_d_*) versus the droplet length at different flow rates and the frequency of the droplet generation.

In the demonstration of single-droplet generation, two ‘input’ modules, a ‘flow-focusing’ module, an ‘observation’ module, and an ‘output’ module are used (see Video S1). The modules are assembled according to the structure shown in Figure 3a. The oil and the deionized water mixed with green solvent dye enter the ‘flow-focusing’ module via two ‘input’ modules. The deionized water used as the discrete phase flows along the main channel, while the oil used as the continuous phase splits into two branches and merges into the main channel. The discretization of the deionized water into droplets occurs at the joint of the main channel and the bifurcated channels. The inset in Figure 3b shows the formation of the droplets in the ‘flow-focusing’ module. The oil squeezes the deionized water from the two sides and then the flow of the deionized water breaks into segments that are equally separated by the oil (see Video S2).

The influence of the flow rate on the formation of the droplets is analyzed. The flow rate of the continuous phase, *Q_c_*, is kept at 10 μL/min and the flow rate of the discrete phase, *Q_d_*, varies within the range of 2 μL/min to 10 μL/min. In the ‘observation’ module, the generated droplets can be observed. As shown in Figure 3c, droplets with different sizes can be generated at different flow rate ratios, i.e., *Q_c_*/*Q_d_*. The droplets are equally spaced along the channel. Small droplets can be generated at a low flow rate of the discrete phase. The relation between the length of the droplets and the flow rate ratio is illustrated in Figure 3d. With the rise of the discrete-phase flow rate, the length of the droplets increases. The variation of the droplet length is in the range of 459 μm to 937 μm. The deviation of the droplet length is within −2% to 2%, indicating the stability of the droplet generation. The frequency is inversely proportional to the flow rate when the flow rate of the continuous phase is fixed. The change in the flow rate mainly affects the size of the droplets and the frequency of the droplet generation.

Droplet length can be represented as $L∼{\left(\mu_{d}/\mu_{c}\right)}^{1/3}{\left(Q_{c}/Q_{d}\right)}^{-1/2}$ , where *μ_d_* (0.001 Pa·s) and *μ_c_* (0.029 Pa·s) are the viscosity of the discrete phase and the continuous phase, respectively. The frequency of the droplet generation is proportional to the droplet length [29]. The measurement of the frequency can be described using a function of $f∼{\left(Q_{c}/Q_{d}\right)}^{-1/2}$. The coefficients of determination are high (*R*^2^ ≥ 0.995) in the curve fitting, indicating that the functions can well predict the droplet size and frequency.

### 3.2. UV Curing of Single Droplets

**Figure 4:**
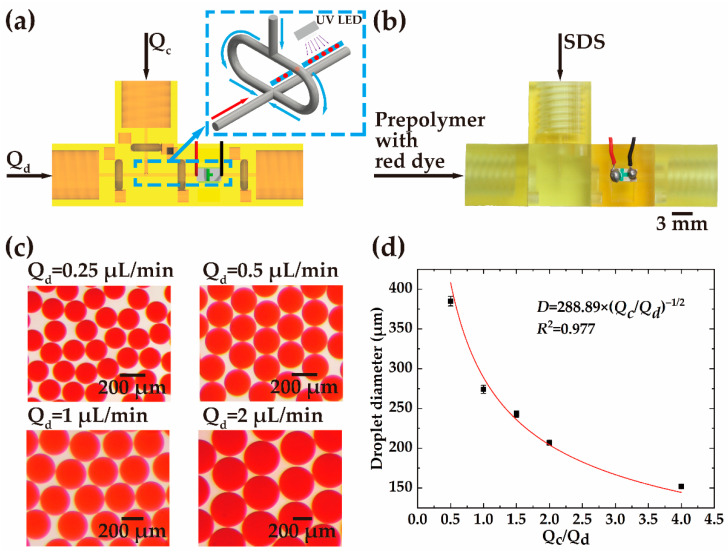
Schematic illustration of generation and UV curing of single droplets. (**a**) Schematic drawing and (**b**) the experimental setup of the assembled system for single-droplet generation and curing. (**c**) The photopolymerized single droplets generated at different flow rates of the discrete phase and the fixed flow rate (1 μL/min) of the continuous phase. (**d**) The plot of the flow rate ratio (*Q_c_*/*Q_d_*) versus the droplet diameter at different flow rates.

To avoid droplet coalescence, UV-curable prepolymer can be added into the discrete phase and then the generated droplets can be photopolymerized under UV exposure. A ‘UV curing’ module is designed with a 365 nm UV LED for UV curing. Figure 4 demonstrates the generation and curing of the single droplets. Two ‘input’ modules are installed on the two sides of a ‘flow-focusing’ module, as shown in Figure 4a. A ‘UV curing’ module follows the ‘flow-focusing’ module. An ‘output’ module is connected to the ‘UV curing’ module. In the experiment, the prepolymer mixed with red dye used as the discrete phase is injected into the main channel, while the SDS solution used as the continuous phase is split into two branches and merged into the main channel (Figure 4b). The formation of the droplets occurs at the joint of the main channel and the bifurcated channels. The curing of the single droplets is realized in the ‘UV curing’ module by irradiation of UV light.

As shown in Figure 4c, the flow rate of the continuous stream (*Q_c_*) is maintained at 1 μL/min, whereas the flow rate of the discrete stream (*Q_d_*) is changed to 0.25 μL/min, 0.5 μL/min, 1 μL/min, and 2 μL/min. The UV dose applied to the droplet (18 mJ) is much higher than the required photocuring dose (2.4 mJ), ensuring the droplet cures. Photopolymerized single droplets of different sizes are obtained after curing. The variation of the droplet diameter is in the range of 152 μm to 385 μm, as shown in Figure 4d. According to the distribution of the droplet size, smaller droplets can be produced if the flow rate of the discrete phase is reduced.

### 3.3. Generation of Alternating Droplets and Merged Droplets

**Figure 5:**
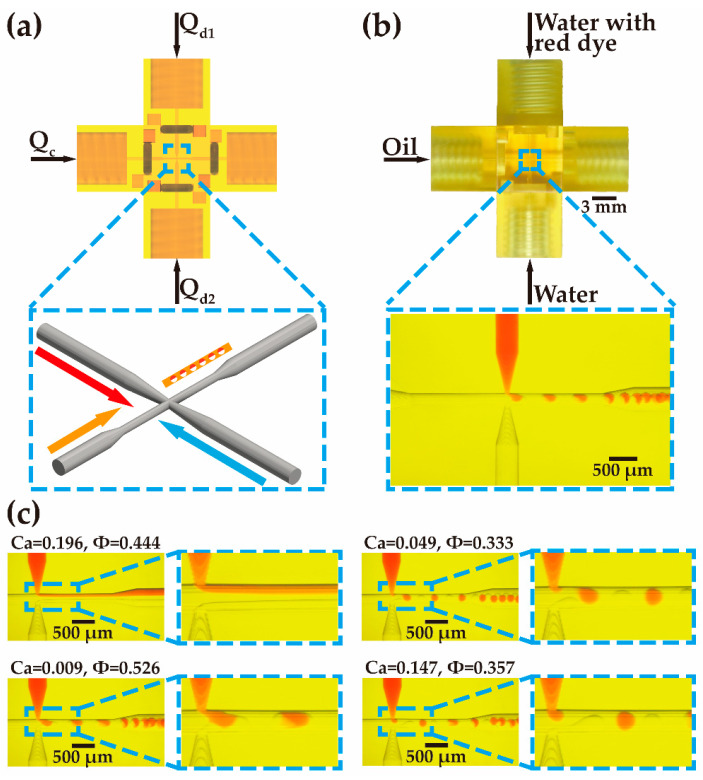
Schematic illustration of alternating-droplet and merged-droplet generation. (**a**) Schematic and (**b**) the printed sample of assembled passive alternating droplet- and merged droplet-generation devices, respectively. (**c**) Optical micrographs of different flow regimes for different values of *Ca* and *Φ*.

To realize the generation of alternating droplets and merged droplets, three ‘input’ modules, a ‘double T-junction’ module, and an ‘output’ module are used. The modules are assembled into a cross-structure, as shown in Figure 5a. The oil is used as a continuous phase and injected into the main channel. The deionized water mixed with glycerol, used as the discrete phase, enters the ‘double T-junction’ module via two ‘input’ modules from the two sides. The deionized water in one of the branches is dyed with a red solvent. The generation of the droplets occurs at the cross of the channels. Figure 5b shows the formation of the droplets in the ‘double T-junction’ module.

As shown in Figure 5c, four states, i.e., laminar flow, alternating droplets, merged droplets, and unstable state can be obtained from the ‘double T-junction’ module with the change of the flow rates of the continuous and discrete phases. In the laminar flow state, two discrete phases smoothly turn the corner from the two side channels into the main channel and then flow along the main channel, keeping to the two sides of the channel. The continuous phase is sandwiched in between the two discrete phases. The continuous and discrete phases are not mixed and flow in parallel, forming laminar flow. With the decrease in the flow rate of the continuous phase, the discrete phases can break into droplets at the cross of the channels. The droplets are generated alternately from the two side channels (see Video S3). The generated droplets are uniform in size and equally spaced. If the flow rate of the continuous phases becomes lower, the droplets from the two side channels bump into each other and form large droplets, then forming merged droplets (see Video S4). In between the alternating droplet and merged droplet states, an unstable state is observed, in which parts of droplets from the side channels coincidentally merge into large droplets while the rest of the individual droplets flow along the main channel.

The formation of the droplets is related to the flow rate fraction and the capillary number of the discrete and continuous phases. The flow rate fraction, *Φ*, between the discrete phase and the continuous phase is defined as

(1) $$\phi =\frac{Q_{d_{1}}+Q_{d_{2}}}{Q_{c}+Q_{d_{1}}+Q_{d_{2}}}$$

where *Q_c_* is the flow rate of the continuous phase, and *Q_d_*_1_ and *Q_d_*_2_ are the flow rates of the two discrete phases. The capillary number, *Ca*, can be defined as

(2) $$\mathrm{Ca}=\frac{\mu_{c}Q_{c}}{\sigma S}$$

where *μ_c_* (0.029 Pa·s) is the viscosity of the continuous phase, *Q_c_* is the flow rate of the continuous phase, *S* is the cross-sectional area of the main channel, and *σ* is the surface tension.

**Figure 6:**
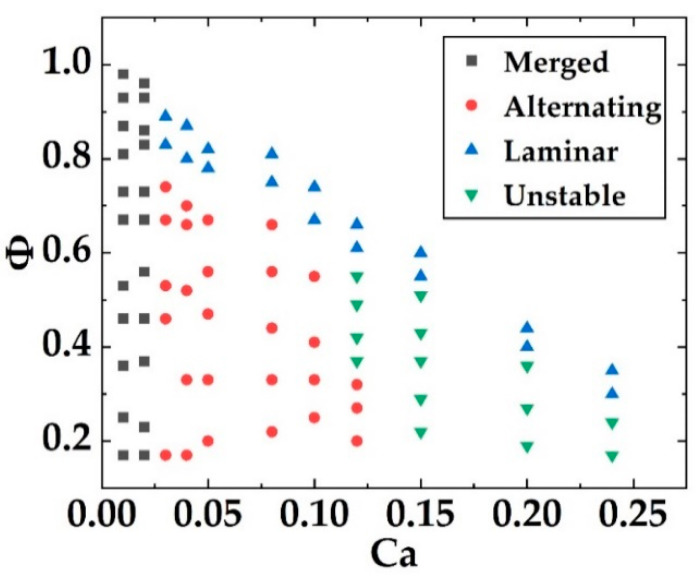
Plot of capillary number, *Ca*, versus flow rate fraction, *Φ*.

Figure 6 plots the statistics of the states under the different conditions of flow rate fraction and capillary number. The laminar flow state can be observed when the capillary number exceeds 0.027 and the flow rate fraction is between 0.28 and 0.89. When the capillary number is high, shear force dominates. When the capillary number is low and the flow rate fraction is high, the continuous phase contributes to a weak shear force, which is not sufficient to break the discrete phase. As a result, the continuous phase flows in between the discrete phases in parallel along the main channel. The alternating droplet state can be realized with the capillary number in the range of 0.027 to 0.12 and the flow rate fraction between 0.2 and 0.74. In the droplet generation, the shear force provided by the continuous phase dominates and acts on the discrete phases, cutting the discrete-phase flow into droplets at the junctions. The merged droplet state is obtained at a low capillary number (0.007 to 0.02) and a flow rate fraction between 0.17 and 0.96. At such a low capillary number of *Ca*, the shear force of the continuous versus the discrete phases is rather low, so it cannot cut the discrete phases at the junction and the merging of discrete phases occurs. Then, after the merging of discrete phases, the increase in the force area leads to the increase in the shear force of the continuous phase so that the combined discrete-phase droplets are formed.

### 3.4. Generation of Janus Particles

**Figure 7:**
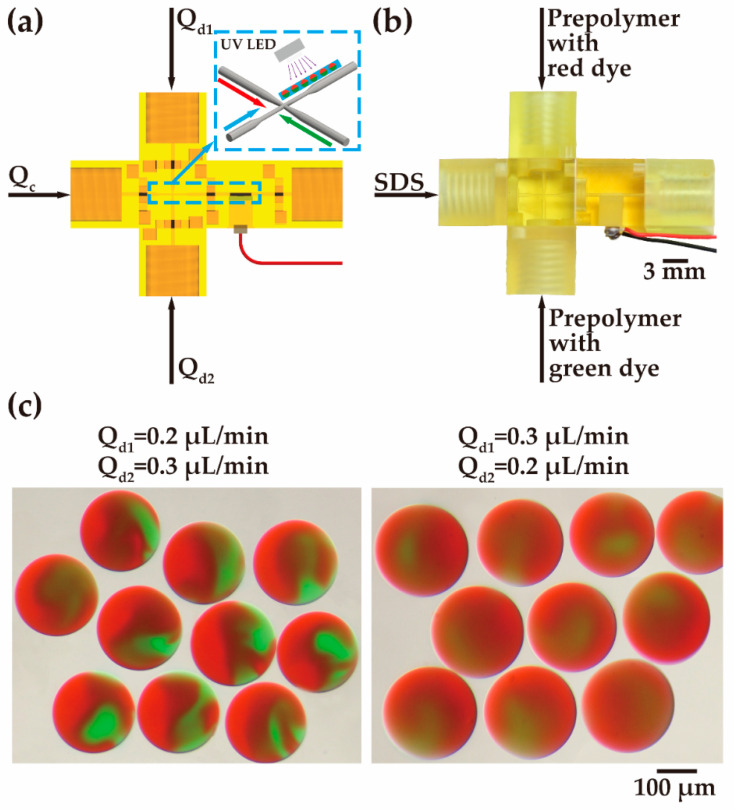
Schematic illustration of Janus particles. (**a**) Schematic drawing and (**b**) the experimental setup of the Janus particle-generation system. (**c**) The generated Janus particles at different flow rates.

The generation of Janus particles is demonstrated. Three ‘input’ modules, a ‘double T-junction’ module, a ‘UV curing’ module, and an ‘output’ module are assembled, as shown in Figure 7a. The SDS solution is used as a continuous phase and injected into the main channel. The prepolymer mixed with red dye and green dye, used as the discrete phase, is injected into the ‘double T-junction’ module via the two ‘input’ modules from the two sides (Figure 7b). The merged droplets are generated in the ‘double T-junction’ module and then solidified in the ‘UV curing’ module, forming Janus particles.

The flow rate of the discrete stream in green (*Q_d_*_2_) and the flow rate of the discrete stream in red (*Q_d_*_1_) vary from 0.2 μL/min to 0.3 μL/min. The continuous stream (*Q_c_*) is kept at 0.8 μL/min. Bi-compartmented Janus particles with different proportions of red and green contents can be achieved (Figure 7c). Thanks to the timely photopolymerization, coalescence of the contents in the two compartments does not happen.

## 4. Conclusions

In conclusion, we propose a standard for designing modules and developing microfluidic systems. In this standard, the size of the modules and the microchannels, the alignment and connection method and fixture structures between the modules, and the directional-flowing strategy are strictly regulated. The microfluidic system can be partially updated and flexibly expanded in a fast and cost-effective way. In the demonstration, a diversity of key modules have been designed based on the standard and produced by 3D printing. We have developed various droplet-generation systems using the standard modules. The modules can be easily cleaned by using ethanol and no contamination occurs. In addition, the surface of the microchannels is originally hydrophobic and can be modified into hydrophilic by simple processing. A variety of droplets have been generated, including single droplets, alternating droplets, merged droplets, and Janus particles. The size of the droplets can be flexibly tuned by controlling the flow rates of the injected streams. The proposed modular microfluidic droplet generator has the potential to be utilized in many biomedical and chemical applications, including single-cell analysis, protein screening, material synthesis, and generation of functional beads. To satisfy the comprehensive requirements in the microfluidic community, we expect more functional modules to be developed for establishing various microfluidic systems.

## Data Availability

The data presented in this study are available on request from the corresponding author.

## Supplementary Materials

The following supporting information can be downloaded at https://www.mdpi.com/article/10.3390/bios12121085/s1, Video S1. Module assembly. Video S2. Single-droplet generation. Video S3. Alternating-droplet generation. Video S4. Merged-droplet generation.
